# Challenges in Implementing Comprehensive Precision Medicine Screening for Ovarian Cancer

**DOI:** 10.3390/curroncol31120592

**Published:** 2024-12-18

**Authors:** Laura R. Moffitt, Nazanin Karimnia, Amy L. Wilson, Andrew N. Stephens, Gwo-Yaw Ho, Maree Bilandzic

**Affiliations:** 1 Hudson Institute of Medical Research, Clayton 3168, Australia; laura.moffitt@hudson.org.au (L.R.M.); naz.karimnia@monash.edu (N.K.); amy.wilson@hudson.org.au (A.L.W.); andrew.n.stephens@monash.edu (A.N.S.); 2 Department of Molecular and Translational Sciences, Monash University, Clayton 3168, Australia; 3 School of Clinical Sciences, Monash University, Clayton 3168, Australia; gwo-yaw.ho@monash.edu; 4 Department of Oncology, Monash Health, Bentleigh 3165, Australia

**Keywords:** precision medicine, epithelial ovarian cancer, tumour heterogeneity, genomic screening, high-throughput drug screening

## Abstract

Precision medicine has revolutionised targeted cancer treatments; however, its implementation in ovarian cancer remains challenging. Diverse tumour biology and extensive heterogeneity in ovarian cancer can limit the translatability of genetic profiling and contribute to a lack of biomarkers of treatment response. This review addresses the barriers in precision medicine for ovarian cancer, including obtaining adequate and representative tissue samples for analysis, developing functional and standardised screening methods, and navigating data infrastructure and management. Ethical concerns related to patient consent, data privacy and health equity are also explored. We highlight the socio-economic complexities for precision medicine and propose strategies to overcome these challenges with an emphasis on accessibility and education amongst patients and health professionals and the development of regulatory frameworks to support clinical integration. Interdisciplinary collaboration is essential to drive progress in precision medicine to improve disease management and ovarian cancer patient outcomes.

## 1. Introduction

The primary challenge in effectively managing ovarian cancer long-term is therapy failure due to recurrence and resistance, which affects up to 90% of patients [1]. While using precision medicine to guide treatment choices has achieved significant success in many cancers including chronic myeloid leukaemia [2], lung [3], and breast cancers [4], only a proportion of ovarian cancer patients are suitable candidates for these targeted approaches due to the unique challenges of ovarian cancer. Consequently, the majority of women with ovarian cancer face limited treatment options, with the emphasis often shifting from curative strategies to palliative care.

Precision medicine traditionally relies on next-generation sequencing (NGS) and the identification of specific genetic markers to guide cancer treatments [5]. However, in the context of ovarian cancer, most genetic alterations are not consistently associated with clinical outcomes [6], which limits the effectiveness of this approach. An exception to this is mutations in the critical homologous recombination (HR) pathway genes BRCA1/2, which are present in only 10–18% of hereditary ovarian cancer cases [7,8]. PARP inhibitors (PARPi) are approved for treating recurrent, advanced BRCA1/2-mutant high-grade serous ovarian cancer and have shown improved overall survival mainly in patients with germline and somatic BRCA1/2 pathogenic mutations or homologous recombination deficiency (HRD) [9], highlighting the importance of these genetic mutations as both prognostic and predictive indicators in a subset of ovarian cancer patients.

Drug repurposing platforms offer the potential to uncover novel therapeutic options for ovarian cancer using high-throughput screening of existing drugs. However, most platforms have predominantly used 2D cell models, which do not accurately reflect the physiology or drug response of ovarian cancer, leading to poor clinical translation [10,11,12]. Given the unique mode of ovarian cancer metastasis, high-throughput drug screening platforms that utilise 3D models such as tumour spheroids or organoids are required. Although various methods have been investigated, a standardised, simple, reliable, and scalable high-throughput 3D screening method remains unavailable [13,14,15].

Therefore, a precision medicine platform that incorporates real-time functional screening offers significant potential to improve cancer treatment outcomes, minimise toxicity, and identify novel therapeutic opportunities; however, these approaches are severely under-utilised and hindered by significant challenges. This review examines the critical infrastructure and resources required to establish a precision medicine platform, explores key limitations such as sample availability, and describes other considerations that must be addressed for its successful implementation for ovarian cancer.

## 2. Ovarian Cancer: A Diverse Spectrum of Malignancies

Ovarian cancer is the sixth leading cause of cancer-related death among women and has the highest mortality rate of any gynaecological malignancy [16]. It is a heterogeneous disease, consisting of three primary subtypes: epithelial, germ cell, and sex cord–stromal, with epithelial ovarian cancer (EOC) being the most prevalent [17]. Most ovarian cancer cases are diagnosed at an advanced stage due to the absence of recognisable symptoms and the lack of effective early detection methods [18]. Although initial treatment involving cytoreductive surgery followed by platinum-/paclitaxel-based chemotherapy is often effective [19], recurrence occurs in 80–85% of patients [20,21]. Despite significant advancements in understanding the molecular landscape of ovarian cancer and the mechanisms driving tumour formation and progression, these insights have not yet led to improved diagnostic techniques or therapies, resulting in a largely unchanged overall survival rate [22]. The management of the disease is further complicated by the inevitable emergence of drug resistance, which contributes to the low 5-year survival rate in advanced-stage ovarian cancer of just 12–30% [23].

Approximately 90% of ovarian tumours arise from epithelial tissue, comprising at least five main subtypes: high-grade serous carcinoma (HGSC), endometrioid carcinoma (EC), clear-cell carcinoma (CCC), mucinous carcinoma (MC), and low-grade serous carcinoma (LGSC). Each histotype is associated with distinct molecular profiles, therapy responses, and clinical presentations as detailed in Table 1 [24].

High-grade serous carcinoma (HGSC) is usually managed with a combination of surgery and platinum-based chemotherapy, followed by maintenance therapy with PARP inhibitors for patients diagnosed with HRD-associated tumours including BRCA1/2 pathogenic mutations [25]. In some cases, low-grade serous carcinomas (LGSCs) are treated with chemotherapy; however, due to the high expression of oestrogen and progesterone receptors in most LGSCs [26,27], these tumours are better managed with hormone blockade therapies such as anastrozole, letrozole, and tamoxifen [28]. Endometrioid and clear-cell carcinomas may benefit from targeted therapies when specific disease mutations are identified, such as immune checkpoint inhibitors for tumours with high microsatellite instability (MSI) [29] or PARP inhibitors for HRD cases [30], while germ cell tumours typically respond well to chemotherapies such as bleomycin, etoposide, and cisplatin [31]. Sex cord–stromal tumours are generally treated with surgery and adjuvant chemotherapy (bleomycin, etoposide, and cisplatin), while radiotherapy is considered in cases of advanced disease or incomplete surgical resection [32].

After initial treatment, HGSC patients are categorised into platinum-resistant (relapse within 6 months) and platinum-sensitive (relapse after more than 6 months) groups, which guide subsequent therapy [33]. For those in the platinum-sensitive group, recurrence is usually managed with platinum-based combination therapies, such as carboplatin paired with gemcitabine, paclitaxel, or pegylated liposomal doxorubicin (PLD), which have shown better outcomes compared to single-agent treatments [34,35]. Additionally, the anti-angiogenic agent bevacizumab has been shown to improve progression-free survival (PFS) from 3.4 to 6.7 months, although it does not significantly increase overall survival (OS) compared to chemotherapy alone [36]. The platinum-resistant group, in contrast, is typically treated with non-platinum chemotherapies, such as oral cyclophosphamide, gemcitabine, paclitaxel, PLD, or topotecan. However, these treatments generally yield poor response rates of 10–15% and a median OS of less than 12 months [37,38,39]. Regardless of platinum sensitivity or treatment strategy, most HGSC patients experience relapse, with progressively diminishing benefits from subsequent therapies.

## 3. Targeted Therapies and Genetic Profiling

Advances in understanding the genetic basis of disease has uncovered new avenues for improving cancer treatment and management, paving the way for targeted therapies and personalised medicines. The development of sequencing and genetic profiling technologies has enabled the identification of actionable mutations and biomarkers, leading to the approval of several drugs that target specific patient characteristics in malignancies such as lung cancer, chronic myeloid leukaemia, multiple myeloma, and breast cancer [40,41].

The concept of targeted treatment involves analysing a patient’s DNA to identify specific mutations or variations that may influence their response to therapies serving as the foundation of conventional precision medicine [5]. In ovarian cancer, tumour biopsies are analysed through DNA extraction and sequencing to identify mutations and variations thatguide treatment decisions [42,43]. For example, gene expression profiling has enabled the stratification of patients with BRCA1/2 gene mutations and other HRDs, allowing this cohort of patients to benefit from targeted personalised treatments such as PARP inhibitors [44]. Similarly in breast cancer, biomarkers such as HER2 have been identified as positive predictors of response to Herceptin (trastuzumab), enabling tailored treatment approaches [45,46]. In cancers such as non-small-cell lung cancer (NSCLC), well-characterised genetic signatures have transformed management with tyrosine kinase inhibitors targeting EGFR mutations and next-generation ALK inhibitors addressing ALK mutations [47], resulting in significantly improved patient outcomes. However, there are several complexities with precision medicine implementation for ovarian cancer; the genetic drivers and dependencies currently remain poorly understood and are largely mediated by epigenetic modifications. Screening for changes in DNA methylation, histone acetylation and chromatin remodelling to reveal novel epigenetic targets represents a promising yet underexplored area, while the absence of universal biomarkers poses a significant challenge hindering the development of standardised precision medicine strategies for this malignancy.

## 4. Personalised Immunotherapy in Ovarian Cancer

Immunotherapy represents a promising advancement in personalised treatment for ovarian cancer and includes the use of immune checkpoint inhibitors, adoptive T-cell therapies, and cancer vaccines. However, the rarity of most ovarian cancer subtypes makes it challenging to identify and validate new biomarkers that could guide the effective use of immunotherapies. For example, while PD-1/PD-L1 inhibitors have shown significant success in other cancers such as melanoma and NSCLC [48,49], their efficacy in ovarian cancer is limited [50], which is complicated by the fact that tumour PD-L1 expression does not reliably predict treatment responsiveness in any of these tumour types [51,52]. As an alternative immunotherapy approach, chimeric antigen receptor (CAR)-T-cell therapy allows for the engineering of patient-derived T-cells to specifically target tumour-associated antigens and offers a highly individualised treatment. CAR-T-cell therapy has been largely effective in haematological malignancies and has shown promise in clinical trials targeting various tumour antigens including HER2, mesothelin, and prostate-specific membrane antigen [53,54,55,56]. However, implementing the CAR-T-cell approach in ovarian cancer is more complex, as the disease represents an immunosuppressive microenvironment, characterised by low immunogenicity and high infiltration of immunosuppressive cells. This reiterates the intricate biological and molecular heterogeneity of ovarian cancer, highlighting the challenges of translating precision medicine approaches into meaningful clinical outcomes for this disease.

Strategies such as combination therapies to enhance T-cell activation or modify the tumour microenvironment are being actively investigated to improve outcomes. While immunotherapy has not yet achieved widespread success in ovarian cancer compared to other malignancies, its potential for personalisation and durable responses underscores its importance in the precision medicine landscape.

## 5. Tumour Heterogeneity: Not a One-Size-Fits-All Approach

Genomic profiling in ovarian cancer has shifted the treatment paradigm towards personalised approaches [57]. However, the limited number of druggable mutations and the extensive heterogeneity observed in ovarian cancer complicates the development of treatment strategies based solely on genetic factors [42,43]. Tumours from different patients, or even distinct cells within the same tumour, often exhibit unique genetic profiles that create variability in treatment outcomes. This inter- and intra-tumoral heterogeneity plays a significant role in disease progression and treatment resistance [58,59].

For example, a study examining 27 tumour biopsies from various metastatic sites in three patients with advanced-stage EOC identified genetic heterogeneity across 400 oncogenes within each sample [60]. Furthermore, the metastatic lesions were genetically distinct not only from the primary tumour but also from other metastatic sites [60]. Similarly, another study involving 31 HGSC samples from six patients, taken before and after chemotherapy, revealed that even well-characterised mutations including PIK3CA were not conserved across all samples, with no consistent mutations or evolutionary patterns observed except for TP53 [61]. Although BRCA1 and BRCA2 mutations are recognised as well-known risk factors for ovarian cancer, they only account for a subset of cases [7,8]. PARP inhibitors such as olaparib effectively target the dysfunctional BRCA1/2 pathways but can also benefit patients with other HRD mutations or epigenetic modulation such as *ATM*, *BARD1*, *MRE11*, *RAD51*, and *PALB2* [9,62], highlighting the complexity of tailoring therapies to the genetic landscape of ovarian cancer. Additionally, current HRD genomic assays face significant limitations, including their inability to provide a functional readout of HR activity or account for tumour evolution. Genomic scars indicating HR deficiency persist even after HR restoration through reversion mutations, which are associated with resistance to PARPi and platinum therapies, undermining the tests’ ability to predict resistance development [63].

The variability in genetic penetrance—the likelihood that a genetic mutation will manifest as disease—further complicates ovarian cancer risk assessment. Even individuals carrying the same genetic mutation may have widely varying risks of developing ovarian cancer [64], making it difficult to establish universal diagnostic and prognostic screening guidelines. Moreover, genetic tests are typically designed to identify specific mutations or pathogenic variants, meaning that mutations outside the tested panels may go undetected. For example, the FDA-approved direct-to-consumer genetic test for inherited BRCA mutations can miss up to 90% of BRCA1/2 mutations associated with cancer [65], underscoring the limitations of current genetic testing strategies.

## 6. Navigating the Limitations of Sample Availability

Precision medicine relies heavily on the availability of high-quality patient samples. However, the relatively low incidence of ovarian cancer presents significant challenges to acquiring sufficient material to generate large databases, develop robust screening protocols, and conduct meaningful analyses. According to the Global Cancer Observatory (GLOBOCAN), countries like Australia, the United Kingdom, and the United States report higher incidence rates, with approximately 10–12 cases per 100,000 women annually [66]. In contrast, Eastern European countries range from 8 to 13 cases per 100,000 women, while countries like Japan and China report lower incidence rates with around 5–8 cases per 100,000 women [66]. These differences may reflect differences in genetic predisposition, dietary habits, diagnostic practices, and reproductive factors.

To address the limitations in sample availability and enhance the capacity for precision medicine, collaboration is key. Coordinated biobanking, cross-institutional collaboration and multi-centre studies with targeted recruitment strategies and prospective sample collection can help to build a more comprehensive repository of samples enabling robust longitudinal studies. This approach is particularly crucial for rare diseases like ovarian cancer, where single institutions often face challenges in gathering sufficient samples. Ovarian cancer biospecimen collection is unique as it includes tumour biopsies from primary and metastatic sites in addition to ovarian cancer cells and fluid derived from malignant ascites, which represent the complex tumour microenvironment. Due to the extensive genetic and molecular diversity present within and between sample types, this makes it difficult to obtain a sample that fully represents the cancer’s complexity [67].

Moreover, the limited sample quantity available after pathological assessment and diagnosis constrains further analyses, such as matched tissue screening [68]. Processing and expanding ascites-derived cells in vitro for screening procedures introduces additional variability as cells cultured on plastic often fail to retain the characteristic signature of the original tumour [69]. These limitations restrict the ability to reliably identify novel therapeutic targets and to uncover the heterogeneity within ovarian cancer subtypes, which is crucial for developing personalised treatment strategies. Sample availability constraints also contribute to small sample sizes, which are a common challenge for data analysis in ovarian cancer research. To ensure the validity of findings, strict statistical methods need to be carefully selected. Non-parametric tests, which do not assume normal data distribution, are well suited to small datasets and provide more reliable results in this context. Effect size calculations, such as Hedge’s g, are also important tools for addressing biases inherent in small sample studies and allow for meaningful comparisons across experiments [70]. Moreover, to strengthen the rigor in these analyses, meta-analytic approaches which combine data from multiple studies can improve power and offer more robust evidence to guide clinical decision-making [71]. Incorporating meta-analytic findings into clinical practice will enhance the translational potential of precision medicine research, helping bridge the gap between laboratory findings and patient care.

High-quality biospecimens are also essential for the generation of accurate datasets for precision medicine research. Optimising pre-analytical factors, including sample collection, preservation, storage, and processing parameters, is essential to ensure the reliability and reproducibility of data [72,73]. For example, the accuracy of data generated from fresh frozen and formalin-fixed paraffin-embedded (FFPE) samples depends heavily on the extraction and storage methods employed [73]. Additionally, obtaining high-quality pre-treatment samples has become more challenging given the increasing use of neoadjuvant chemotherapy for ovarian cancer [74]. Poor sample quality or variability in processing can introduce confounding factors or bias, directly impacting research findings and their clinical implication. Given the extensive heterogeneity observed in ovarian cancers, the use of high-quality biospecimens is therefore essential so as not to introduce further variations in the data. Furthermore, implementing rigorous quality assurance checks can help maintain sample integrity, making it easier to compare datasets and draw meaningful conclusions [68,75].

Future advancements must prioritise strategies to maximise sample utilisation and improve the efficiency of sample collection. Developing sensitive assays that require smaller sample volumes and enhancing biobanking practices to preserve sample integrity over extended periods are essential steps forward. Continued efforts to develop robust protocols and methods for sample collection, processing, and storage are also required to maintain the integrity and viability of the samples. Additionally, centralised multi-institutional collaborations and centralised biobanks can pool resources to increase sample availability, enabling more comprehensive studies. Achieving these goals will require addressing the substantial resource demands associated with sample collection, processing, and storage while ensuring the availability of sufficient, high-quality samples.

## 7. Functional Screening Constraints

Despite significant advances in NGS technology for precision medicine, it is increasingly evident that genomics alone is insufficient to accurately predict drug responses [76]. Functional drug screening offers a more complete approach by integrating genomic insights with functional assays that capture the biological processes driving disease progression [77]. This combination provides a robust framework for evaluating drug responses in the context of the disease’s complexity. However, functional screening faces critical challenges particularly in developing in vitro assays that generate reliable and disease-relevant drug responses while maintaining high-throughput capacity.

Indeed, high-throughput drug repurposing platforms hold significant promise for identifying new therapeutic opportunities in ovarian cancer management. These platforms are extensively employed to profile large compound libraries, using end-point cytotoxicity as a key output [78]. While effective for initial drug screening, these pipelines are frequently constrained by their reliance on 2D cell culture models. Monolayer cultures, though convenient for high-throughput applications, fail to replicate the physiological state or drug sensitivity of ovarian cancer [11,12]. For example, cells in 2D cultures are typically more sensitive to lower treatment doses in viability assays compared to the in vivo environment, leading to results that may not translate effectively to clinical settings. By contrast, 3D culture models, which better mimic tumour architecture and the tumour microenvironment, offer a more physiologically relevant platform for functional drug screening. However, their implementation requires additional technical expertise, specialised infrastructure, and resources to scale for high-throughput applications.

Limited sample availability often necessitates the use of “cherry-picked” drug panels for functional screening. These panels typically focus on established or emerging treatments, targeting well-established pathways. However, this restricted approach risks overlooking drugs with novel or less-understood mechanisms of action or beneficial off-target effects, particularly those targeting less explored or unknown pathways. Although intended to streamline the initial screening process, cherry-picking significantly narrows the scope of discovery. This bias restricts the scope of discovery, potentially omitting innovative therapeutic candidates and reinforcing existing assumptions. For ovarian cancer, a disease characterised by significant heterogeneity and complexity, this narrowed focus may lead to an incomplete understanding of the therapeutic landscape and missed opportunities for identifying transformative treatments.

Another limitation of high-throughput screening is the use of a single drug concentration or a limited number of drug concentrations, which can result in a high rate of false positives or negatives. This approach does not provide insights into whether the observed effects are due to excessively high drug concentrations or a lack of efficacy at lower doses, thereby failing to capture the full dose–response spectrum [79,80]. As a result, drugs with moderate potency but favourable therapeutic windows may be overlooked. This issue is particularly critical when seeking to identify effective combination therapies, as the exclusion of certain compounds may limit the discovery of synergistic effects. While secondary screens integrating dose–response curves can address this limitation, incorporating a range of concentrations into the primary screen would be beneficial. However, this requires a greater amount of starting material, posing a significant challenge when working with limited clinical samples.

Addressing these constraints requires the development of innovative methodologies and collaborative strategies. Expanding the use of 3D culture models, improving sample preservation techniques, and leveraging computational and artificial intelligence (AI) tools to predict synergistic effects could enhance the efficiency and relevance of functional screening. Additionally, the integration of multiplexed assays and the exploration of underrepresented drug classes may provide a broader understanding of ovarian cancer’s therapeutic landscape. By overcoming these challenges, functional screening can fulfil its potential to drive the discovery of novel, effective treatments for ovarian cancer and other complex diseases.

## 8. Requirements for Complex Resources, Data Infrastructure, and Management

Precision medicine screening relies on advanced technologies, such as NGS, high-throughput screening (HTS) instrumentation, and sophisticated imaging platforms. These technologies are essential for accurately identifying genetic variations, screening large libraries of compounds and visualising biological processes in high detail. Although the cost of genetic technologies has decreased significantly over the years [81], the acquisition, maintenance, and operation of such equipment are still cost-prohibitive, particularly for smaller institutions and laboratories that may lack the financial resources to invest in these tools. To mitigate these costs, researchers often turn to fee-for-service providers. However, this approach may require bulk sample processing to manage expenses, which complicates timely analysis and limits the ability to refine and iterate experimental design in real time.

Precision screening approaches generate vast quantities of genetic, clinical, and experimental data. A critical aspect of this is the integration and standardisation of electronic health records (EHRs), which are often incomplete, fragmented, or not designed for high-throughput research. Australia’s National Cancer Data Framework (Cancer Australia) represents a significant step forward in enhancing the collection, integration, and utilisation of cancer-related data. While this initiative is commendable, it is essential that the framework incorporates biobanking services to maximise its potential impact. To address these limitations, there should be seamless linkage between clinical data and biospecimen repositories, while research protocols should be carefully crafted by multidisciplinary teams to ensure consistency and data integrity [82]. Furthermore, the analysis of large datasets requires bioinformatics expertise, supported by intelligent data management systems and interdisciplinary tools accessible across all personnel involved in the precision medicine pipeline.

Developing robust infrastructure to store, process, and analyse these data demands investment in hardware, data pipelines, and specialised algorithms. These systems must be tailored to the specific needs of precision medicine projects and involve technical expertise that often extends beyond the initial funding scope of the project. To alleviate these challenges, government investment is crucial to subsidise costs for consumers. Partnerships between private and public sectors can also enhance accessibility, ensuring that advanced services are available to both researchers and patients.

Additionally, there are logistical workflow disruption challenges; adjusting to these approaches in the clinic necessitates new protocols for patient assessment, data interpretation, and therapeutic decision-making, which may not align seamlessly with traditional methods. This can strain already-overburdened healthcare systems, particularly in settings with limited staff or resources.

The multidisciplinary nature of precision medicine further necessitates a diverse and highly skilled workforce, encompassing laboratory-based molecular biologists, pathologists, bioinformaticians, data scientists, and clinical oncologists [83]. Each of these roles requires specialised training and expertise, increasing the resource demands of such initiatives and highlighting the need for ongoing upskilling to keep pace with research advancements. Maintaining full-time staff for projects with infrequent sample collection presents significant operational and financial challenges. In addition, sustaining operational continuity is challenging when sample collection and screening activities are sporadic and would delay project milestones and budgets. To circumvent these constraints, forming strategic partnerships could provide access to additional expertise and share the burden of staffing and resource allocation. For example, joint research fellows could contribute to the screening of cancers with multiple origins, optimising the utilisation of their expertise. Moreover, the establishment of a coordinated international service dedicated to ovarian cancer sample acquisition could greatly expand the capacity and scope of screening platforms.

## 9. Ensuring Ethical Compliance

Implementing precision medicine screening into clinical practice raises a range of ethical challenges, particularly concerning how results are communicated to patients. These challenges encompass issues related to informed consent, the interpretation of complex data, the psychological impact of findings, and the broader implications of acquiring and utilising genetic information. One critical concern is addressing health literacy disparities and education gaps between different patient cohorts. An ongoing challenge in the willingness of patients to participate in precision medicine screening procedures is the lack of adequate education about what precision medicine entails, including the potential discovery of incidental findings or results with uncertain clinical significance. Patients must fully understand what they are consenting to, highlighting the need for clear and comprehensive communication about the scope, risks, and limitations of these procedures [84]. Another challenge lies in the interpretation of genomic variants, particularly of those with unknown significance. Miscommunication or inadequate explanation of these findings can lead to confusion, anxiety, or even misguided medical decisions. Effective communication between laboratory researchers and clinicians is essential to ensure accurate interpretation and clear explanations of complex data to patients [85]. This collaboration is particularly crucial when dealing with sensitive genetic information, as misinterpretation or overinterpretation can lead to confusion and anxiety for patients, resulting in unnecessary stress.

Privacy and data security are also paramount concerns. With reluctance surrounding the potential for the misuse of genetic information, patients need to be reliably informed on how it is protected and how discrimination is mitigated [84]. For instance, the fear of genetic discrimination often deters patients from undergoing genetic testing, particularly due to its impact on life insurance. In Australia, new legislation will ban life insurers from using genetic test results to deny cover or increase premiums. This comprehensive protection aligns with precedents in Canada and the UK, aiming to encourage genetic testing and advance personalised medicine. However, while announced, the legislation is not yet in effect [86]. Transparent policies on data storage, sharing, and security are essential to build trust and encourage participation in precision medicine initiatives.

Furthermore, while in vitro drug screening provides essential insights into potential therapeutic options, it is important to recognise the risk of “false hope”. This phenomenon occurs when promising drug targets identified in the laboratory fail to translate into effective patient outcomes [87]. The gap between laboratory findings and clinical efficacy can lead to significant disappointment for patients who might have been led to believe that a new treatment option was possible. Managing patient expectations is therefore critical to minimising emotional distress and maintaining trust in the healthcare process.

Additionally, ensuring the timely translation of patient data to a feasible treatment remains a significant challenge. A recent study on the clinical translation of paediatric precision medicine determined the average turnaround time from enrolment (sample collection) to clinical reporting was almost 9 weeks [88]. During this timeframe, various factors need to be managed including ongoing treatment/monitoring, the risk of disease progression, and the unfortunate case where research findings may not provide useful insights to improve patient outcomes. These delays and uncertainties underscore the importance of streamlining workflows and managing patient care effectively while research progresses. Ensuring ethical compliance in precision medicine requires addressing the unique challenges of informed consent, data interpretation, patient education, privacy, and the psychological impact of findings. Clear communication, robust privacy protection, and realistic management of patient expectations are crucial for building trust and ensuring the responsible implementation of precision medicine in clinical practice.

## 10. Integration with Clinical Practice: Social and Economic Considerations

One significant barrier to integrating precision medicine into clinical practice is the lack of large-scale evidence demonstrating its efficacy, compounded by the absence of well-designed clinical trials to support the benefits of precision medicine in diverse populations [84]. Recruiting sufficient participants for ovarian cancer trials, in particular, is challenging due to its relatively low incidence and patient treatment histories where patients do not meet eligibility criteria or may be too unwell to participate [89]. Evidence suggests there is also a clear knowledge gap in the clinical advantages of precision medicine amongst various healthcare professionals (oncologists, pharmacists, etc.) [84,90], including diagnostic issues with precision medicine-based approaches based on clinical misinterpretation.

Although off-label drug use is common in oncology, clinicians require additional education on tailoring treatments based on individual patient profiles, including accessibility, regulatory, and cost–benefit risks [91,92]. A curated approach involving the integration of genomic, clinical, lifestyle, and other factors will further assist with decision-making and clinical recommendations for precision medicine [93]. Robust clinical trial data alongside improved education and multidisciplinary collaboration will significantly enhance the acceptance and adoption of precision medicine by clinicians [94].

The financial burden of precision medicine is another critical challenge, affecting both patients and healthcare systems [95]. For example, comprehensive tumour profiling, laboratory testing, and the reporting of clinically relevant recommendations back to the patient are estimated to cost over $20,000 per patient [88]. For rare diseases such as ovarian cancer, assessing cost effectiveness is complex as the expenses associated with diagnostics and personalised treatments may outweigh the savings by the lower burden on our healthcare system. Strengthening economic modelling is essential to assess the relative cost effectiveness of precision medicine approaches. Additionally, healthcare schemes and subsidies must be developed to support personalised medicine and alleviate financial pressures on patients.

Moreover, comprehensive genetic testing and other precision medicine approaches are not accessible to all patients, particularly those in underserved or low-resource settings, limiting the effectiveness of precision medicine on a broader scale. Language barriers can also exacerbate health equity gaps; a 2016 study by the Royal Australian College of General Practitioners found that 16% of patients in general practice consultations spoke a language other than English (LOTE), but only 5% of all healthcare consultations involved communicating in an LOTE [96], whilst many healthcare resources are not translated beyond English.

Cultural sensitivity is a critical consideration in the implementation of precision medicine, as trust in the healthcare system varies significantly across different communities. Historical injustices, such as unethical medical practices or exclusion from research, have led to deep-rooted mistrust among some groups, particularly First Nations communities and ethnic minorities [97]. This mistrust can hinder participation in genetic testing, clinical trials, and biobanking, which are foundational to precision medicine. Additionally, cultural beliefs about health, illness, and genetics may influence how patients perceive and engage with precision medicine. Effective communication that respects cultural values, addresses concerns transparently, and involves trusted community leaders is essential to build trust.

Furthermore, datasets derived from precision medicine may not reflect diversity within the population, which increases the risk of discrimination and can exacerbate existing healthcare inequalities [98]. This lack of representation in previous and ongoing genomic studies and datasets, such as the Cancer Genome Atlas (TCGA; https://www.cancer.gov/tcga (accessed on 13 November 2024)) and Genome Aggregation Database (gnomAD; https://gnomad.broadinstitute.org (accessed on 13 November 2024)), contributes to disparities in understanding genetic variations across different populations. For instance, variants of unknown significance (VUS) are more frequently observed in individuals of African descent compared to those of European ancestry [99]. One study showed that African American women with breast cancer were more than 2.5 times as likely to carry at least one VUS compared to White women (40% vs. 14.5%) [100]. This disparity is rooted in the underrepresentation of diverse populations in genetic studies, leading to a lack of comprehensive reference data for interpreting genetic variants in non-European groups. The increased prevalence of VUS in Black populations hinders the clinical utility of genetic testing by leaving many findings ambiguous, which can delay or complicate risk assessment and management strategies.

Addressing these barriers through education, equitable access, culturally sensitive practices, and diverse data collection will be critical for the broader adoption and effectiveness of precision medicine.

## 11. Alternative and Complementary Approaches to Functional Precision Medicine for Ovarian Cancer

Alternative approaches to functional precision medicine for ovarian cancer should aim to address its heterogeneity through multiple innovative tools and techniques. Multi-omics integration combining genomics, transcriptomics, proteomics, metabolomics, and epigenomics could offer a holistic understanding of ovarian cancer biology, uncovering novel actionable pathways and biomarkers for use in downstream precision medicine approaches. Implementing these advanced methods in routine clinical workflows demands specialised bioinformatics expertise to analyse and interpret large, complex datasets, alongside the high costs associated with generating and processing multi-omics data. Moreover, the variability in data quality and interpretation standards and the challenge of integrating findings into actionable clinical decisions further complicate their adoption. Nonetheless, this approach represents an exciting frontier in precision medicine screening.

CRISPR-based functional screens may identify key vulnerabilities, while AI and machine learning can enhance the power of multi-omics by identifying patterns in complex datasets to predict treatment responses and optimise patient stratification for clinical trials. Proteomic and metabolomic analyses add crucial insights into active signalling pathways and metabolic dependencies, while spatial transcriptomics can offer a detailed view of the tumour microenvironment. Patient-derived organoids and xenograft models may additionally serve as biologically relevant platforms for testing drug responses. By integrating these methods with systemic barriers, precision medicine for ovarian cancer can be significantly enhanced, enabling more effective and personalised treatment strategies for this disease.

## 12. Conclusions

**Figure 1:**
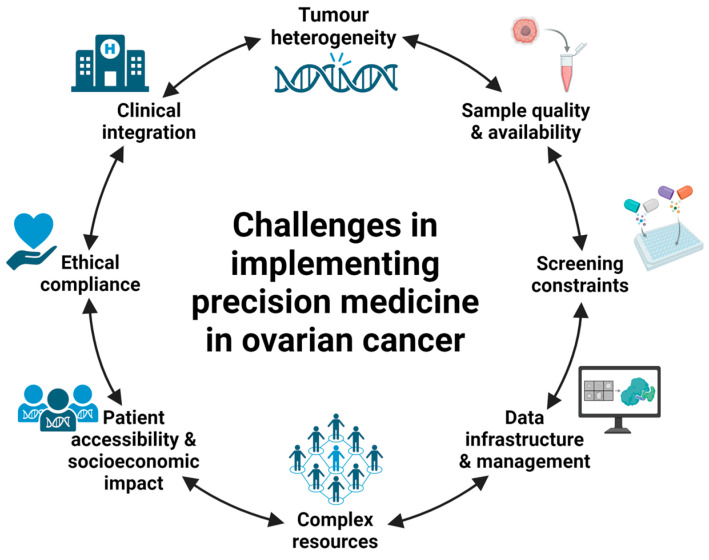
A summary of the challenges associated with implementing precision medicine screening for ovarian cancer samples. Created with BioRender.com.

Precision medicine holds significant potential to transform the treatment and outcomes of ovarian cancer, but its successful integration into clinical practice requires addressing several critical challenges (outlined in Figure 1). Tumour heterogeneity, limited sample availability, the absence of reliable functional screens, and the lack of robust clinical trial data create substantial barriers to the development and implementation of effective, personalised therapies. Additionally, safeguarding patient data is essential to maintain privacy and trust in precision medicine incentives.

To address the obstacles faced by precision medicine, interdisciplinary collaboration is paramount. Multidisciplinary teams, including clinicians, researchers, bioinformaticians, and policymakers, must work together to close knowledge gaps, improve infrastructure, and advance precision medicine approaches. Equally important is the need for enhanced education for both healthcare professionals and patients, ensuring all stakeholders are well informed to navigate the complexities of precision medicine.

Moreover, it is essential to focus on preventing healthcare inequalities by ensuring that advancements in precision medicine are accessible to diverse patient populations including those in underserved regions. Policymakers must prioritise the development of comprehensive frameworks to support the ethical, equitable, and practical implementation of precision medicine. These policies should address economic, social, and regulatory challenges while promoting inclusivity in research and clinical applications.

By overcoming these challenges, precision medicine can achieve its full potential, improving the standard of care for ovarian cancer patients and offering hope for better outcomes in this challenging and heterogeneous disease.

## Figures and Tables

**Table 1:** Clinical properties of EOC histotypes.

	HGSC	EC	CCC	MC	LGSC
Percentage of cases	75%	10%	6%	<5%	<5%
Most common stage at presentation	Advanced	Early	Early	Early	Advanced
Response to first-line treatment	Sensitive	Sensitive	Resistant	Resistant	Resistant
5-year survival rate	32.1%	44.7%	22.3%	13.9%	54.2%
Molecular aberrations	P53, BRCA1/2, HRD, PI3K amplification	PTEN, ARID1A, MSI	PI3K mutation, ARID1A, MSI	KRAS, HER2	BRAF, KRAS, NRAS
